# Patients with Pandemic (H1N1) 2009 in Intensive Care Units, Israel

**DOI:** 10.3201/eid1604.091696

**Published:** 2010-04

**Authors:** Eran Kopel, Ziva Amitai, Itamar Grotto, Ehud Kaliner, Irina Volovik

**Affiliations:** Ministry of Health, Tel Aviv, Israel

**Keywords:** Influenza, pandemic (H1N1) 2009, human, intensive care units, public health, virus, Israel, expedited, letter

**To the Editor:** We report results of an active surveillance system established by the Tel Aviv District Health Office in Israel. This surveillance system monitors the daily status of patients with laboratory-confirmed pandemic (H1N1) 2009 virus infection in each of the district’s intensive care units (ICUs), including pediatric ICUs.

Follow-up is maintained by daily phone conversations with medical staff until disease outcome is concluded by discharge, transfer to a long-term rehabilitation facility, or death. Medical records, as well as daily laboratory reports, are collected to confirm or to rule out pandemic (H1N1) 2009 infection.

During July 10–October 10, 2009, our prospective cohort included 17 patients with pandemic (H1N1) 2009 laboratory-confirmed infection who were residents of the district; 12 (70.6%) were male patients. The median age was 44 years (interquartile range 13–72 years). By October 10, 2009, six patients had been discharged, 7 had died, 2 had been transferred to long-term rehabilitation facilities, and 2 remained hospitalized.

Twelve (70.6%) patients had an underlying medical condition, mainly chronic lung disease (6 patients) or chronic cardiovascular disease (5 patients). Two patients were morbidly obese (body mass index >35), and 1 patient was pregnant. Additionally, 3 patients (17.6%) were infected while hospitalized.

Thirteen patients (76.5%) had acute respiratory distress syndrome caused by diffuse viral pneumonitis. Other notable manifestations were acute renal failure (6 patients), sepsis/septic shock (5 patients), and neurologic complications such as Guillain-Barré syndrome, encephalitis, and seizures (3 patients).

Documented nosocomial sepsis, often of multiple gram-negative bacteria (9 patients), was the most frequent complication during the course of the disease. Other frequent characteristics were the use of high positive end-expiratory pressure during mechanical ventilation (4 patients) and the need for tracheostomy (5 patients).

Average time from disease onset to hospital admission was 3 days. Time from hospital admission to ICU admission for those patients who died was longer than for those who survived, with a median of 2 days compared with 0.5 day, respectively, albeit not significant (p = 0.26). Average hospitalization was 23.4 days; average length of stay in the ICU was 16.7 days (71.4% of the average hospitalization time).

As mentioned previously, 7 patients (41.2%) died; 5 (71.4%) were male, similar to their cohort’s proportion. One significant difference (p = 0.02) was found between the age of survivors (mean 26.0 years, 95% confidence interval 7.6–44.3) and the age of nonsurvivors (mean 59.3 years, 95% confidence interval 39.6–79.0). The most prominent case–fatality rate was for elderly patients, >65 years of age (3 of 4 patients) followed by patients between 20 and 64 years of age (4 of 9 patients); these subgroups constituted 23.5% and 52.9% of the cohort, respectively.

Estimated incidence rate was 13.8 patients and 5.7 deaths in ICUs per million residents in the Tel Aviv district. Again, the elderly subgroup was dominant, with the highest estimated rate of illness (23.1 per million residents) and death rate (17.3 per million residents). The denominator of these rates was calculated from the population data published by the Israeli Central Bureau of Statistics for 2007 and 2008. Upon that basis, the population data for the end of the third quarter of 2009 was estimated.

During the described surveillance period, 5.7% of ICU beds in the district were, on average, continuously occupied by patients infected with pandemic (H1N1) 2009. The occupancy peak was 6.5 of 53.8 standardized ICU beds (12.1%) per million residents during the week ending August 28, 2009 (Figure).

**Figure:**
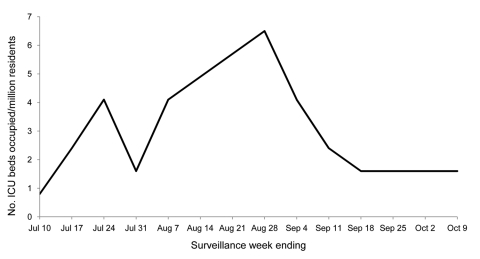
Number of intensive care unit (ICU) beds occupied by patients with pandemic (H1N1) 2009 infection in district ICUs during the described surveillance period, Tel Aviv, Israel. During this period, 5.7% of ICU beds, on average, were continuously occupied by patients with pandemic (H1N1) 2009 infection. The occupancy peak was 6.5 of 53.8 standardized ICU beds per million residents (12.1%) during the week ending August 28, 2009. Data are per million residents.

In conclusion, our analysis of patients having the most severe pandemic (H1N1) 2009 infection indicates a need for prolonged periods of hospitalizations, especially in ICUs, for young adults and elderly patients. Death or prolonged adverse complications were frequent outcomes. We found that the impact of patients with pandemic (H1N1) 2009 on the ICUs in our district during the summer wave was surprisingly similar in length and intensity to the impact that was recently reported in Australia and New Zealand during the winter wave (*1*). The maximum number of ICU beds occupied per million residents, reported for all regions of Australia and New Zealand combined, was 7.4 during the week ending July 27, 2009 (vs. 6.5 as described above). We also found that the mean age of those who died was older than that in previous reports (*2**–**6*).This finding may present a need for policymakers to reconsider current vaccination priorities (*7*) while facing the winter wave of influenza in the Northern Hemisphere.
